# YRNA expression predicts survival in bladder cancer patients

**DOI:** 10.1186/s12885-017-3746-y

**Published:** 2017-11-10

**Authors:** Yuri Tolkach, Anna Franziska Stahl, Eva-Maria Niehoff, Chenming Zhao, Glen Kristiansen, Stefan Cajetan Müller, Jörg Ellinger

**Affiliations:** 1 Institute of Pathology, University Hospital Bonn, Bonn, Germany; 2 Department of Urology, University Hospital Bonn, Bonn, Germany

**Keywords:** YRNA, Biomarker, Bladder cancer, Prognosis

## Abstract

**Background:**

Non-coding RNAs play an important role in human carcinogenesis. YRNAs (Ro-associated Y), a novel class of non-coding RNAs, have been identified as biomarker in various malignancies, but remain to be studied in urinary bladder cancer (BCA) patients.

**Methods:**

The expression of all four YRNAs (RNY1, RNY3, RNY4, RNY5) was determined in archival BCA (urothelial carcinoma, *n* = 88) and normal urothelial bladder (*n* = 30) tissues using quantitative real-time PCR. Associations with clinicopathological parameters and prognostic role for overall and cancer-specific survival were analysed.

**Results:**

All YRNAs were significantly downregulated in BCA tissue. A low expression of RNY1, RNY3 and RNY4 was associated with muscle-invasive BCA, lymph node metastases and advanced grade. Furthermore, expression of RNY1 and RNY3 was predictive for BCA patients’ overall (also RNY4) and cancer-specific survival as estimated using Kaplan-Meier and univariate (but not multivariate) Cox regression analyses. RNY1, RNY3 and RNY4 show good discriminative ability between tumor and normal tissue, as well as between muscle-invasive and non-muscle-invasive urothelial carcinoma.

**Conclusions:**

The expression of YRNAs is altered in BCA and associated with poor prognosis. Possible diagnostic role of YRNAs should be investigated in further studies.

**Electronic supplementary material:**

The online version of this article (10.1186/s12885-017-3746-y) contains supplementary material, which is available to authorized users.

## Background

Urinary bladder cancer (BCA) is among the most common malignancies worldwide; approximately 430.000 new cases and 165.000 deaths were estimated for 2012 [1]. An important step in BCA progression is the invasion of the detrusor muscle and metastatic spread. BCA symptoms are sometimes non-specific leading to delayed diagnoses at an invasive stage, which is accompanied with an unfavorable outcome. To improve the therapeutic management a better understanding of the molecular biology of BCA is necessary.

The vast majority of the human genome (98%) consists of non-coding genes [2]. Non-coding RNA (ncRNAs) do not encode proteins, but have a putative regulative function of gene expression. The ncRNAs are classified according to their size in nucleotides (nt) into small-ncRNAs (sncRNA <200 nt) and long-ncRNAs (lncRNA >200 nt) [3]. Much effort has been spent to identify and functionally characterize dysregulated microRNAs [4, 5] and lncRNAs [6] in BCA in the past years, but few is known about other subtypes of the ncRNAs. YRNAs (Ro-associated Y) were recognized as a component of soluble ribonucleoproteins (Ro RNPS) in the blood of patients with rheumatic autoimmune diseases [7]. Nowadays, four highly conserved human YRNAs (RNY1, RNY3, RNY4, and RNY5) are known. YRNAs have a size of 80–110 nt and a stem-loop structure due to their complementary 5′ and 3′ ends [8]. They are functionally relevant for DNA replication [9] and Ro60 inhibition [10]. YRNAs are overexpressed in various cancer cells [11], and RNY1 and RNY3 inhibition was shown to decrease cell proliferation [11, 12]. YRNA-derived fragments are involved in caspase-3-dependent cell death and NF-κB-dependent inflammation and may have an inflammatory role [13]. It was also shown that RNY5 fragments in extracellular vesicles trigger cell death, and thereby may help cancer cells to optimize the microenvironment for proliferation and invasion [14]. YRNAs have not been investigated in a large cohort of BCA so far; we therefore studied the expression profile of YRNAs in BCA and normal urothelial tissue.

## Methods

### Patients

Formalin-fixed, paraffin embedded (FFPE) bladder tissues were randomly selected from the archive of the Institute of pathology at the University Hospital Bonn from patients (*n* = 112) who underwent transurethral resection of the bladder (TURB) or radical cystectomy for BCA from 1990 until 2009. Follow-up information was available for all patients; median follow-up time was 51 months (range 1–210). The detailed clinicopathological parameters are reported in Table 1.

**Table 1:** Clinicopathological characteristics of the study cohort

	Normal	BCA
	*n* = 30	*n* = 88
Sex		
Male	22 (73%)	70 (79.5%)
Female	8 (27%)	18 (20.5%)
Age		
Mean	66.3	69.1
Range	43–81	40–91
Staging pT-stage		
pTa	n.a.	13 (14.8%)
pTis	n.a.	13 (14.8%)
pT1	n.a.	18 (20.5%)
pT2	n.a.	12 (13.6%)
pT3	n.a.	15 (17.0%)
pT4	n.a.	17 (19.3%)
Lymph node metastasis	n.a.	23 (26.1%)
Distant metastasis	n.a.	2 (2.3%)
Grading		
grade 1	n.a.	9 (10.2%)
grade 2	n.a.	31 (35.2%)
grade 3	n.a.	48 (54.5%)

### Ethics, consent and permissions

All patients gave written informed consent for the collection of biomaterials within the framework of the Biobank at the University Hospital Bonn. The study was approved by the ethic committee (280/12) at the University Hospital Bonn.

### Tissue samples acquisition

A first 5 μm thick section from the FFPE block was stained with haematoxylin and eosin and used for histological control and mapping of the block content. BCA (*n* = 88) and normal urothelial tissue (*n* = 30) samples were then macrodissected using a scalpel from five consecutive 20 μm sections of the block. The absence of significant inflammation as well as absence of any signs of dysplasia/atypia was ensured morphologically in normal tissues. Some samples with normal urothelial tissue stemmed from patients with BCA, when spatial divergence of these samples could be guaranteed and carcinoma in situ did not coexist.

### RNA isolation and quantitative real-time PCR

The RNA was isolated using the Recover All Total Nucleic Acid Isolation Kit (Ambion, Foster City, CA, USA) according to the suppliers recommendations. Afterwards, the DNA-free DNA Removal Kit (Ambion) was used to digest DNA contaminants. RNA purity and concentration were determined using the NanoDrop 2000 spectrophotometer (Thermo Scientific, Wilmington, DE, USA). The isolated total RNA was stored at −80 °C until further use.

cDNA synthesis and real-time PCR were performed as described in detail by Nientiedt et al. [12], adopted for FFPE tissues. In brief, reverse transcription was carried out with the miScript II RT Kit (Qiagen, Hilden, Germany). Then, 1 ng cDNA template was used for quantification using the Qiagen miScript SYBR Green PCR technology (Hilden, Germany). Self-designed primers were used for the quantification of YRNAs (RNY1 forward: GGC-TGG-TCC-GAA-GGT-AGT-GAG; RNY1 reverse: GGG-GGA-AAG-AGT-AGA-ACA-AGG; RNY3 forward: CCG-AGT-GCA-GTG-GTG-TTT-AC; RNY3 reverse: AAG-CAG-TGG-GAG-TGG-AGA-A; RNY4 forward: TCC-GAT-GGT-AGT-GGG-TTA-TCA; RNY4 reverse: AAA-GCC-AGT-CAA-ATT-TAG-CAG-T. The primer design was performed using Primer-BLAST [15]. The RNY5 primer was published by Christov et al. [9].. YRNA expression levels were normalized to SNORD43 (Qiagen miScript Primer Assay: MS00007476) and RNU6–2 (Qiagen miScript Primer Assay: MS00007476), earlier shown to be a suitable reference gene for urological malignancies [5, 16]. PCR experiments were carried out on a Quant Studio 5 Real-Time PCR System (Applied Biosystems, Foster City, CA, USA). Relative YRNA expression levels were calculated using the 2^-∆∆CT^ algorithm; the PCR efficiencies were: RNY1 101.0%, RNY3 95.3%, RNY4 95.7%, RNY5 104.7%. Each PCR assay included multiple control samples (no-RT-sample, genomic DNA, no template control, RT4 cell line RNA as positive control).

### Statistics

Statistical analyses were performed R (R Foundation for Statistical Computing; version 3.3.3). The Mann-Whitney-Wilcoxon test was used to compare YRNA expression in subgroups. The Spearman-Rho test was used to correlate clinical parameters and YRNA expression. Kaplan-Meier curves, log-rank test, univariate and multivariate Cox proportional hazards regression analysis were used for survival analyses. pROC-package for R was used for ROC-Analyses. survMisc-package was used for selection of the best cutoff during survival analyses.

## Results

**Fig. 1:**
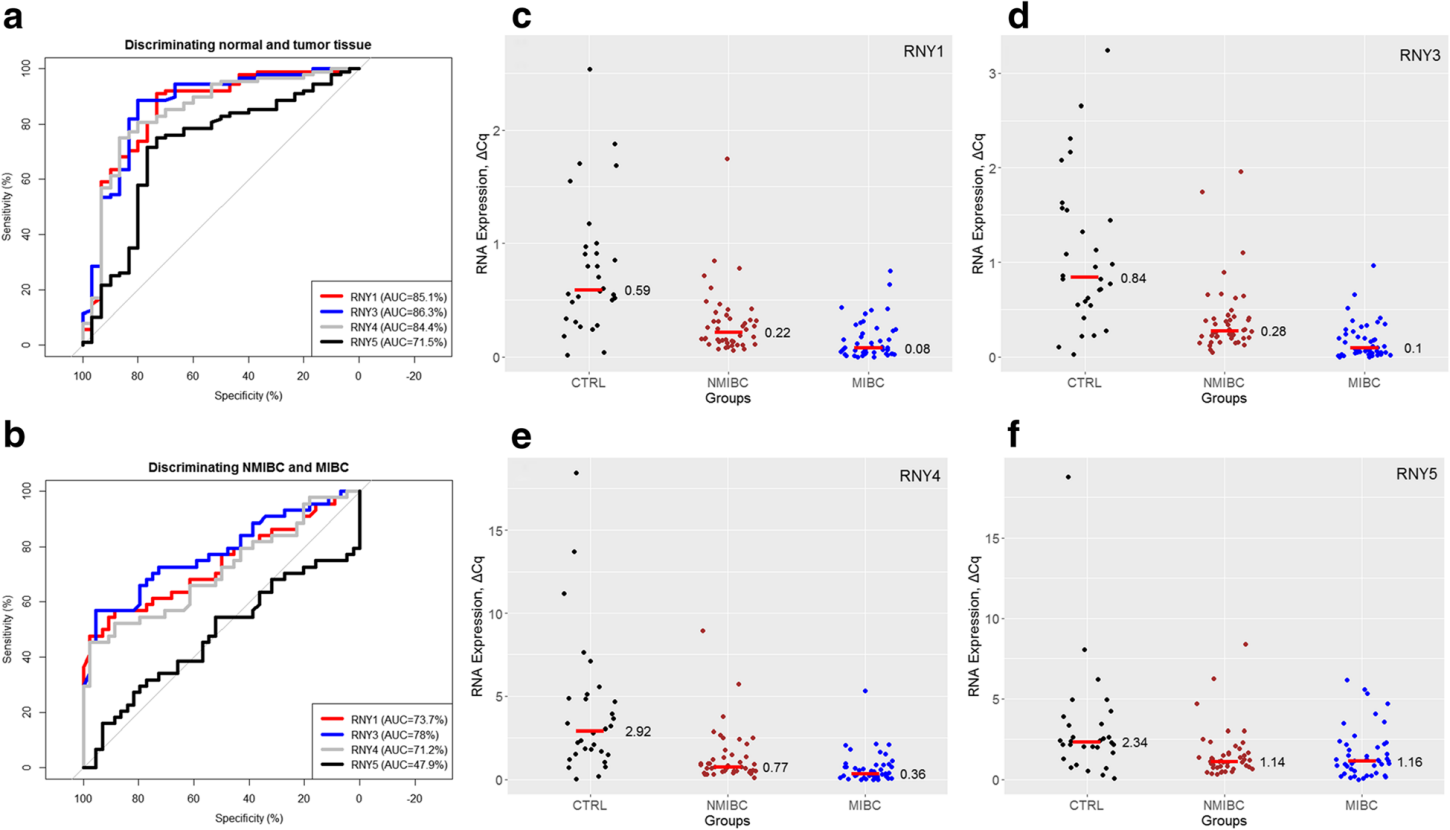
The expression of YRNAs (ΔCq Expression) was determined in a cohort of 30 normal urothelial (CTRL) and 88 bladder cancer (BCA) tissue samples. All YRNAs were significantly downregulated in BCA (all *p* < 0.001, see Table 2). **a** Receiver operator characteristic (ROC) analysis for YRNA-expression to discriminate between normal (CTRL) and tumor (BCA) tissue. **b** ROC-analysis for YRNA-expression to discriminate between muscle-invasive (MIBC) and non-muscle invasive bladder cancer (NMIBC). **c**–**f** RNY1-, RNY3-, RNY4- and RNY5-expression in normal, MIBC and NMIBC tissue samples (p-level < 0.001 for RNY1, RNY3 and RNY4; p-level = 0.739 for RNY5). Short horizontal red line with number = Expression median

Firstly, we have compared the YRNA expression levels in normal and malignant bladder tissues. Median expression levels (Table 2) were significantly (all *p* < 0.001) lower in BCA tissue than in normal tissue (RNY1: 0.59 in normal vs 0.15 in BCA; RNY3: 0.84 in normal vs 0.21 in BCA; RNY4: 2.92 in normal vs 0.61 in BCA; and RNY5: 2.34 in normal vs 1.15 in BCA). As determined using ROC analyses (Fig. 1a, Table 2), the tissue analysis of YRNAs allowed discrimination of BCA tumor tissue and normal urothelial mucosa with an area under curve of 0.715 (RNY5) up to 0.863 (RNY3). YRNA expression levels were correlated with each other (all *p* < 0.001, Additional file 1: Figure S1): especially RNY1, RNY3 and RNY4 expression was highly correlated (r^2^ > 0.87), whereas RNY5 levels were less distinctly correlated to RNY1 (r^2^ = 0.45), RNY3 (r^2^ = 0.40) and RNY4 (r^2^ = 0.66).

**Table 2:** Relative YRNA expression levels in the tumor and normal urothelial tissue and discriminative capabilities of the YRNAs to predict the tissue dignity (tumor vs. normal)

	Expression median (range)		ROC analysis		Sensitivity	Specificity	Cut-off
	BCA (*n* = 88)	CTRL (*n* = 30)	AUC	95%CI			
RNY1	0.15 (0.0–1.75)	0.59 (0.02–2.54)	0.851	0.760–0.941	73.3%	90.9%	0.471
RNY3	0.21 (0.0–1.96)	0.84 (0.03–3.24)	0.863	0.778–0.949	80.0%	88.6%	0.527
RNY4	0.61 (0.0–8.96)	2.92 (0.05–18.41)	0.844	0.755–0.933	86.7%	75.0%	1.061
RNY5	1.15 (0.0–8.41)	2.34 (0.07–18.75)	0.715	0.601–0.829	73.3%	75.0%	1.948

Comments: BCA – tissue samples with urothelial cancer (bladder cancer); CTRL – control samples (normal urothelial tissue); AUC, area under the curve; 95%CI, 95% confidence interval

Furthermore, we analyzed, whether differences in YRNA expression were associated with adverse clinicopathological parameters (Additional files 2, 3 and 4: Tables S1–S3). RNY1-, RNY3- and RNY4-expression (all *p* < 0.001) was significantly decreased in muscle-invasive BCA (MIBC) compared to non-muscle-invasive BCA (NMIBC), whereas RNY5 expression levels in those were similar (*p* = 0.739) (Fig. 1c–f). Discrimination of the MIBC and NMIBC tumors was possible with maximal AUC of 0.780 for RNY3 (Fig. 1b). The expression of RNY1 (*p* = 0.011), RNY3 (*p* < 0.001) and RNY4 (*p* = 0.041) was also lower in high grade (G3) compared to lower grade (G1 and G2) tumors; RNY5 (*p* = 0.877) expression levels were not correlated with grading. Presence of lymph node metastases was also associated with decreased RNY1 (*p* < 0.001), RNY3 (*p* < 0.001) and RNY4 (*p* = 0.007) expression. YRNA expression was not correlated with age nor gender (all *p* > 0.2).

**Fig. 2:**
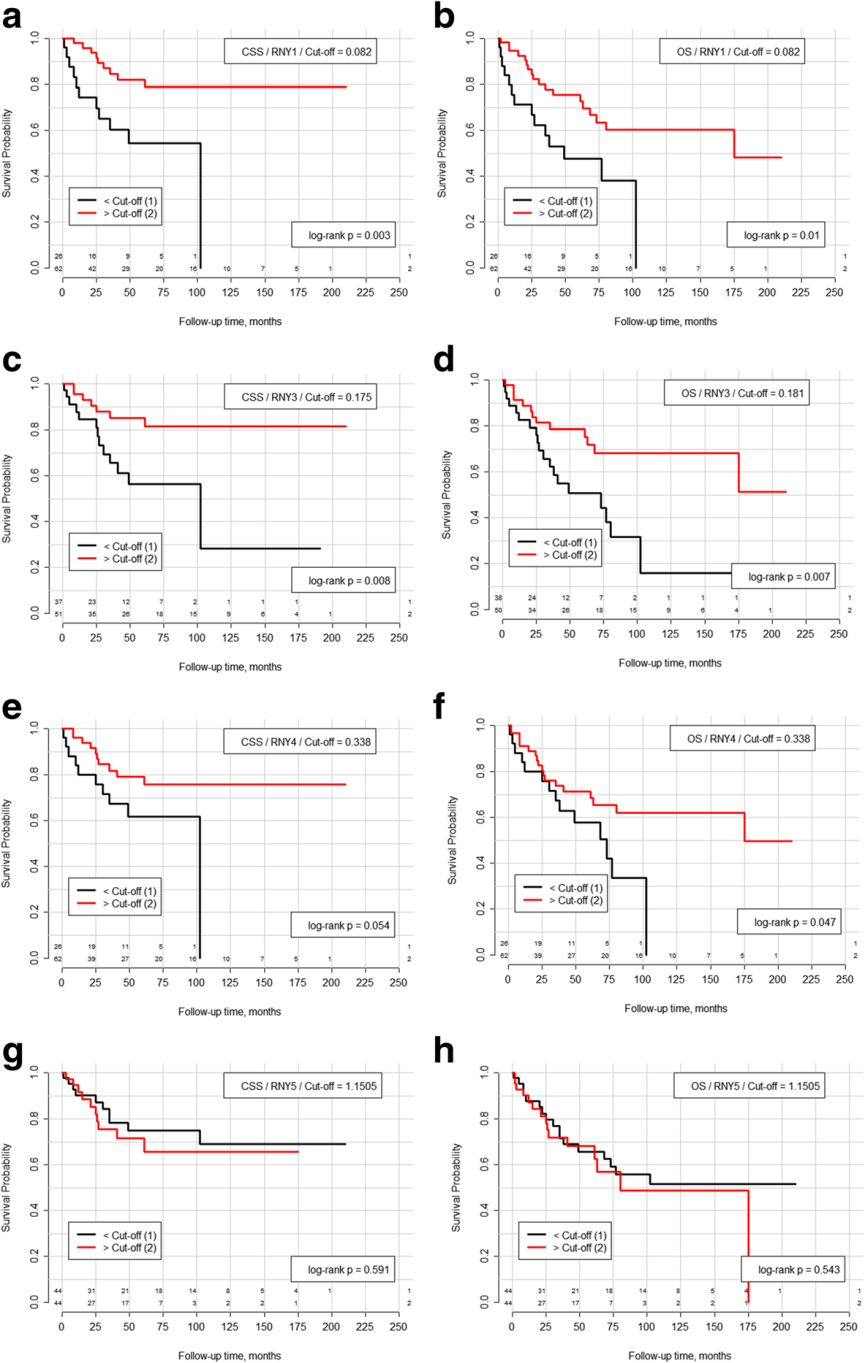
Kaplan-Meier curves and log-rank test for YRNAs expression dichotomized based on the best cut-off, in each case separately for cancer-specific and overall survival, respectively: **a**/**b** RNY1, **c**/**d** RNY3, **e**/**f** RNY4, and **g**/**h** RNY5. Kaplan Meier estimates indicate that expression of YRNAs is statistically significant prognostic for cancer-specific survival (RNY1, RNY3) and overall survival (RNY1, RNY3, RNY4) in BCA patients (all log-rank *p* > 0.05). Abbreviations: OS – overall survival, CSS – cancer-specific survival

Finally, the relevance of YRNAs for patients’ prognosis was determined using Kaplan Meier estimates. Expression of YRNA was significantly correlated with BCA patients’ overall (RNY1, RNY3, RNY4) and cancer-specific (RNY1, RNY3) survival (all log rank *p* < 0.05; Fig. 2). We also performed univariate and multivariate Cox regression analyses: RNY1 and RNY3 were significantly predictive for cancer-specific and overall survival (all *p* < 0.05), but lost their predictive value in a multivariate model (see Tables 3 and 4 for details).

**Table 3:** Cox regression analysis for the prediction of cancer-specific survival in patients with urothelial bladder cancer (*n* = 88, number of events = 20)

	Univariate analysis			Multivariate analysis^a^		
	*p*-value	HR	95% CI	*p*-value	HR	95% CI
RNY1, low vs high	0.005	3.57	1.46–8.72	0.676	1.22	0.47–3.17
RNY3, low vs high	0.012	3.29	1.29–8.35	0.715	1.20	0.45–3.25
RNY4, low vs high	0.062	2.34	0.96–5.69	0.816	0.90	0.35–2.27
RNY5, low vs high	0.593	0.79	0.32–1.90	0.494	0.73	0.30–1.80
MIBC vs. NMIBC	6.7e-04	12.89	2.95–56.26	0.017	7.97*	1.45–43.71
pN-stage, pN1 vs pN0	0.001	4.38	1.81–10.59	0.741	1.19*	0.43–3.29
cM-stage, M1 (*n* = 2) vs M0	2.9e-04	21.2	4.06–110.8	x	x	x
Grade, G3 vs G1 + G2	0.006	4.72	1.57–14.21	0.347	1.82*	0.52–6.35

Comments: ^a^In case of every YRNA data is shown for separate multivariate model with “MIBC vs NMIBC”, pN-Stage and Grade as co-variates; *HR, *p*-value, 95% CI shown for multivariate model with RNY1. Other YRNAs (RNY3, RNY4, RNY5) showed similar results

Analysis for YRNAs is based on the best cut-off, the same as in Kaplan-Meier/log-rank analysis: RNY1–0.082, RNY3–0.175, RNY4–0.338, RNY5–1.151. *Abbreviations*: *MIBC* muscle-invasive bladder cancer, *NMIBC* non-muscle-invasive bladder cancer

**Table 4:** Cox regression analysis for the prediction of overall survival in patients with urothelial bladder cancer (*n* = 88, number of events = 32)

Overall Survival	Univariate analysis			Multivariate analysis^a^		
	*p*-value	HR	95% CI	*p*-value	HR	95% CI
RNY1, low vs high	0.013	2.49	1.22–5.08	0.615	1.24	0.55–2.80
RNY3, low vs high	0.009	2.60	1.27–5.33	0.400	1.43	0.62–3.26
RNY4, low vs high	0.053	2.03	0.99–4.15	0.847	1.08	0.49–2.41
RNY5, low vs high	0.545	0.80	0.40–1.63	0.531	0.80	0.39–1.62
MIBC vs. NMIBC	4.9e-04	4.05	1.85–8.90	0.055	2.86*	0.98–8.36
pN-stage, pN1 vs pN0	0.003	2.89	1.44–5.84	0.641	1.24*	0.50–3.05
cM-stage, M1 (n = 2) vs M0	0.001	12.87	2.71–61.16	x	x	x
Grade, G3 vs G1 + G2	0.021	2.39	1.14–5.01	0.575	1.29*	0.55–3.09

Comments: ^a^In case of every YRNA data is shown for separate multivariate model with “MIBC vs NMIBC”, pN-Stage and Grade as co-variates; *HR, *p*-value, 95% CI shown for multivariate model with RNY1. Other YRNAs (RNY3, RNY4, RNY5) showed similar results

Analysis for YRNAs is based on the best cut-off, the same as in Kaplan-Meier/log-rank analysis: RNY1–0.082, RNY3–0.181, RNY4–0.338, RNY5–1.151. *Abbreviations*: *MIBC* muscle-invasive bladder cancer, *NMIBC* non-muscle-invasive bladder cancer

## Discussion

YRNAs have been identified as novel non-coding class of RNA molecules which may be used as biomarker for cancer [17–19]. So far, little information exists about the expression of YRNA in BCA patients. In 2008, Christov et al. demonstrated an increase of YRNA expression [11]. However, his study cohort was very small (*n* = 4) and thereby limiting any meaningful statistical conclusion. Thus, we investigated the expression of all four YRNAs in an enlarged cohort of BCA patients to allow a robust statistical analysis. Interestingly, YRNA expression levels were significantly downregulated in our dataset, mean expression levels in BCA tissue were 2- to 4-fold lower than in normal tissue. It should be noted that Christov et al. [11] normalized the YRNA expression to the mRNA HPRT1, whereas our study used RNU6–2 and SNORD43; RNU6–2 and SNORD43 were earlier shown to be useful reference genes for the analysis of BCA samples [16]. Notably, as expected from the experiments of Christov et al. [11], RNY1, RNY3 and RNY4 expression was highly correlated, whereas the degree of correlation of RNY5 to the other YRNAs was less pronounced.

The analysis of 88 tissue samples with urothelial carcinoma allowed as to correlate the expression of YRNAs with clinicopathological parameters. RNY1, RNY3 and RNY4 expression was associated with advanced stage (muscle invasive BCA, lymph node metastasis) and grade (G3 tumors when opposed to G1 and G2). Importantly, the expression levels of RNY1 and RNY3 were significantly predictive for cancer-specific and overall survival of BCA patients with a clear trend for RNY4. However, the strong correlation of YRNA expression and muscle-invasiveness of the tumor impaired achieving an independency in the multivariate Cox regression analyses within a cohort of 88 BCA patients.

Although YRNA are non-coding RNAs, they are also of functional relevance and do not represent transcriptory garbage. YRNAs are essential factors for chromosomal DNA replication [9], whereby they execute their function during the initiation of DNA replication [20]. siRNA mediated knock-down of RNY1 and RNY3 reduced the number proportion of S phase cells in the HeLa cells; degradation of RNY3 reduced also the number S-phase cells in EJ30 bladder cancer cells. Furthermore, the mitotic index and the cell density was reduced after treatment with RNY3 siRNAs [11]. Within this context it is interesting that we have observed a decrease of YRNA levels in BCA patients. Seemingly, Similar trends with decreased abundance of several YRNAs in tumor patients (in serum) were observed in other tumor types (head and neck squamous cell carcinomas [18], breast cancer [19]), which support our findings, even given the fact that conclusions from cell line studies are suggesting the upregulation could be associated with tumor growth and proliferation This could be related, from one side, to different YRNA effects in different primary tumors and, from the other side, to the artificial construct and well known limitations of cell cultures. Also, the effect of YRNA overexpression was not studied in the above mentioned cell culture study [11]. The biological functions of the YRNA are still understudied and could be multidirectional. Some studies show that YRNAs demonstrating decreased levels during mitosis and high levels during S and G2 phases of the cell cycle, partially through association with chromatin [21]. This may be a possible explanation for decreased expression in highly proliferating tumor tissues. Many microRNAs are known for “managing” the cell fate and cell proliferation through interactions with p53 and other members of p53-family [22], which is highly deregulated in tumor tissue of patients with urinary bladder cancer compared to normal tissue [23]. The interactions with this pathway were not studied for YRNA to date and would probably also provide the explanations for aberrant YRNA expression. It may also be speculated that YRNAs are secreted by BCA cells to act as mediator of immunoescape: extracellular YRNAs fragments activate TLR7 to promote apoptosis in macrophages and monocytes [13].

YRNAs are expected to be a suitable non-invasive biomarker because approximately 25 to 33 nt large YRNA fragments have been identified using small RNA sequencing in human serum and plasma [17]. It was further shown that changes of specific YRNA fragments in serum are associated with ER-negative breast cancer [19]. Similarly, specific YRNA fragments were also circulating at altered levels in head and neck cancer patients [18]. However, specific identification of these small (25 to 33 nt size) fragments implies application of small RNA sequencing procedures and is therefore at least today not suited for daily routine. In our study we were able for the first time to show that RNY1, RNY3 and RNY4 could very good discriminate between the normal and tumor tissue with a maximal AUC of 0.863 (RNY3), and to a lesser extent between muscle-invasive and not-muscle-invasive tumors (maximal AUC 0.780 for RNY3). These findings could support the diagnostic value of YRNA, which certainly warrants further investigations.

Some limitations of our study should be acknowledged: The RNA integrity was not determined after RNA isolation. Formalin-fixed, paraffin embedded tissues are usually degraded to approximately >200–400 bp sized RNA fragments [24, 25], and thus amplifying PCR products of approximately 100 bp size is feasible. We randomly picked samples obtained over a period of approximately 20 years and long-term storage may alter the RNA integrity [26]. However, relative YRNA expression levels were not correlated with the year of surgery (data not shown). The normal urothelial tissue samples were in several patients derived from patients with BCA, however necessary precautions were undertaken to prevent contamination of normal samples with tumor tissue (see Materials and methods). Although even in this case we cannot exclude molecular alterations occurred in the microscopically normal urothelium. The tissue was macrodissected with a scalpel, thus RNA some degree of inevitable contamination with other cells like inflammatory, stromal or endothelial cells could have affected the YRNA expression studies.

## Conclusions

The expression of all four YRNAs is downregulated in tumor tissue in patients with urinary bladder urothelial carcinoma. Expression changes are associated with advanced disease, higher grade and metastatic disease and may have prognostic relevance for cancer-specific and overall survival.

## Additional files

Additional file 1: Figure S1. Correlation matrix (Spearman rho) between the expression of different YRNAs (PCR, ΔCq Expression) in tumor tissue (all *p* < 0.001). (DOCX 29 kb)

Additional file 2: Table S1. Expression of RNYs (PCR, ΔCq expression): non-muscle-invasive bladder cancer (NMIBC) vs muscle-invasive bladder cancer (NMIBC). (DOCX 15 kb)

Additional file 3: Table S2. Expression of RNYs (PCR, ΔCq expression): patients with lymph node metastases (pN+) vs patients without lymph node metastases (pN0). (DOCX 15 kb)

Additional file 4: Table S3. Expression of RNYs (PCR, ΔCq expression): patients stratified according to grade of tumor differentiation. (DOCX 15 kb)

## Abbreviations

BCA: Bladder cancer
FFPE: Formalin-fixed, paraffin embedded
ncRNA: non-coding RNA
YRNA: Ro-associated Y RNA
