# Supplementary material for: YRNA expression predicts survival in bladder cancer patients
Source: BMC Cancer. 2017 Nov 10;17:749. doi: 10.1186/s12885-017-3746-y (PMC5681827; doi:10.1186/s12885-017-3746-y)

**Additional file 1: Figure S1**

Correlation matrix (Spearman rho) between the expression of different YRNAs (PCR, ΔCq Expression) in tumor tissue (all p<0.001).


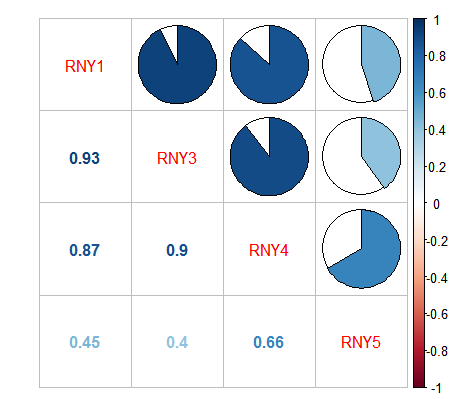

Supplement: Supplementary file 1 — Correlation matrix (Spearman rho) between the expression of different YRNAs (PCR, ΔCq Expression) in tumor tissue (all p < 0.001). (DOCX 29 kb) [file 12885_2017_3746_MOESM1_ESM.docx]
