# Supplementary material for: YRNA expression predicts survival in bladder cancer patients
Source: BMC Cancer. 2017 Nov 10;17:749. doi: 10.1186/s12885-017-3746-y (PMC5681827; doi:10.1186/s12885-017-3746-y)
Supplement: Supplementary file 2 — Expression of RNYs (PCR, ΔCq expression): non-muscle-invasive bladder cancer (NMIBC) vs muscle-invasive bladder cancer (NMIBC). (DOCX 15 kb) [file 12885_2017_3746_MOESM2_ESM.docx]

**Additional file 2: Table S1**

Expression of RNYs (PCR, ΔCq expression): non-muscle-invasive bladder cancer (NMIBC) vs muscle-invasive bladder cancer (NMIBC)

|  | **NMIBC (n=44)** | **MIBC (n=44)** | **p-level*** |
| --- | --- | --- | --- |
| **RNY1**, median (range) | 0.22 (0.06-1.75) | 0.08 (0.0-0.76) | <0.001 |
| **RNY3**, median (range) | 0.28 (0.05-1.96) | 0.1 (0.0-0.97) | <0.001 |
| **RNY4**, median (range) | 0.77 (0.12-8.96) | 0.36 (0.0-5.34) | <0.001 |
| **RNY5**, median (range) | 1.14 (0.34-8.41) | 1.16 (0.0-6.18) | 0.739 |

*Mann-Whitney-Wilcoxon Test
