# Supplementary material for: YRNA expression predicts survival in bladder cancer patients
Source: BMC Cancer. 2017 Nov 10;17:749. doi: 10.1186/s12885-017-3746-y (PMC5681827; doi:10.1186/s12885-017-3746-y)
Supplement: Supplementary file 3 — Expression of RNYs (PCR, ΔCq expression): patients with lymph node metastases (pN+) vs patients without lymph node metastases (pN0). (DOCX 15 kb) [file 12885_2017_3746_MOESM3_ESM.docx]

**Additional file 3: Table S2**

Expression of RNYs (PCR, ΔCq expression): patients with lymph node metastases (pN+) vs patients without lymph node metastases (pN0)

|  | **pN0 (n=65)** | **pN+ (n=23)** | **p-level*** |
| --- | --- | --- | --- |
| **RNY1**, median (range) | 0.18 (0.0-1.75) | 0.04 (0.0-0.41) | <0.001 |
| **RNY3**, median (range) | 0.24 (0.0-1.96) | 0.05 (0.0-0.51) | <0.001 |
| **RNY4**, median (range) | 0.69 (0.0-8.96) | 0.26 (0.0-2.14) | 0.007 |
| **RNY5**, median (range) | 1.21(0.0-8.41) | 1.15 (0.07-6.18) | 0.835 |

*Mann-Whitney-Wilcoxon Test
