# Supplementary material for: YRNA expression predicts survival in bladder cancer patients
Source: BMC Cancer. 2017 Nov 10;17:749. doi: 10.1186/s12885-017-3746-y (PMC5681827; doi:10.1186/s12885-017-3746-y)
Supplement: Supplementary file 4 — Expression of RNYs (PCR, ΔCq expression): patients stratified according to grade of tumor differentiation. (DOCX 15 kb) [file 12885_2017_3746_MOESM4_ESM.docx]

**Additional file 4: Table S3**

Expression of RNYs (PCR, ΔCq expression): patients stratified according to grade of tumor differentiation

|  | **G1 (n=9)** | **G2 (n=31)** | **G3 (n=48)** | **p-level*  (G3 vs G1/2)** |
| --- | --- | --- | --- | --- |
| **RNY1**, median (range) | 0.15 (0.07-0.42) | 0.24 (0.02-0.85) | 0.11 (0.0-1.75) | 0.011 |
| **RNY3**, median (range) | 0.22 (0.08-0.67) | 0.29 (0.03-1.75) | 0.12 (0.0-1.96) | <0.001 |
| **RNY4**, median (range) | 0.67 (0.12-2.52) | 0.89 (0.0-5.34) | 0.54 (0.0-8.96) | 0.041 |
| **RNY5**, median (range) | 1.31 (0.48-2.50) | 1.06 (0.15-8.41) | 1.15 (0.0-6.24) | 0.877 |

*Mann-Whitney-Wilcoxon Test
